# Decoding the molecular landscape of the placenta in maternal diabetes: a systematic review of high-throughput data

**DOI:** 10.1530/JME-25-0131

**Published:** 2026-04-10

**Authors:** Georgia Fakonti, Abigail R Byford, Eleanor M Scott, Beth Holder, Karen Forbes

**Affiliations:** ^1^ Discovery and Translational Science Department, Leeds Institute of Cardiovascular and Metabolic Medicine, University of Leeds, Leeds, United Kingdom; ^2^ Clinical and Population Sciences, Leeds Institute of Cardiovascular and Metabolic Medicine, University of Leeds, Leeds, United Kingdom; ^3^ Institute of Reproductive Biology, Imperial College London, London, United Kingdom

**Keywords:** gestational diabetes mellitus, type 1 diabetes mellitus, type 2 diabetes mellitus, placenta, high-throughput, omics, RNA sequencing, proteomics, functional enrichment analysis

## Abstract

Diabetes in pregnancy is associated with significant short- and long-term complications for mothers and offspring, many of which are thought to result from altered placental development and function. Although studies have demonstrated molecular changes in the placenta in this context, the precise mechanisms remain unclear. High-throughput transcriptomic and proteomic approaches provide powerful tools to systematically identify disease-associated pathways, yet no systematic synthesis of this literature has been undertaken. We conducted a systematic review of omics studies examining placental molecular changes in pregnancies complicated by diabetes compared with uncomplicated pregnancies. Fifty-six studies were eligible for inclusion, the majority of which focused on gestational diabetes mellitus (GDM; *n* = 52). Of these, 42 reported changes in RNA (*n* = 30) or protein (*n* = 12) abundance, with eight proteins and 189 RNA species consistently altered in at least two studies. Functional enrichment analysis revealed dysregulation of immune, vascular, and developmental pathways. Notably, 98 molecules were altered at both RNA and protein levels, 47 with consistent directionality across studies, suggesting robust disruption of core biological pathways. Comparisons across diabetes types showed partial overlap of differentially expressed transcripts between GDM and type 1 diabetes (16 genes) and GDM and type 2 diabetes (34 genes), although no universal markers were identified. These findings highlight shared molecular signatures in GDM, provide novel insights into pathways linking maternal diabetes to placental dysfunction and adverse outcomes, and emphasise the need for further studies on type 1 and type 2 diabetes. These pathways may represent potential therapeutic targets to mitigate intergenerational cardiometabolic risk.

## Introduction

Diabetes in pregnancy is one of the most common pregnancy complications (1), with gestational diabetes mellitus (GDM) being the most prevalent form, affecting 13–26% of pregnancies worldwide (2). Approximately 87.5% of all diabetes cases in pregnancy are GDM, while type 1 and type 2 diabetes mellitus (T1DM and T2DM), along with other less common types, contribute to the remainder (3). Although GDM typically resolves after childbirth, it substantially increases the risk of future cardiometabolic diseases for both mother and offspring, including the development of T2DM post-partum (4, 5, 6). Similar patterns of increased risk of cardiometabolic risk are seen in pregnancies complicated by T1DM and T2DM (7). Cardiac changes in both the mother and fetus, as well as changes in fetal adiposity, have been reported across diabetic pregnancies, emphasising the potential for gestational exposures to influence life-long health trajectories (8, 9, 10, 11, 12, 13, 14). Reducing these risks requires a clear understanding of the biological mechanisms linking maternal diabetes with altered pregnancy and long-term health outcomes.

Fetal development depends on a finely regulated balance between maternal and fetal nutritional demands, with the placenta serving as the central interface that mediates nutrient and oxygen transfer, waste removal, and endocrine communication (15, 16). Numerous studies have reported alterations in placental development and function in pregnancies complicated by diabetes; however, findings are often conflicting. Many investigations document changes in nutrient transport capacity, mitochondrial function, inflammatory signalling, vascular development, and syncytiotrophoblast turnover, yet the direction and magnitude of these changes vary across studies (17, 18, 19, 20, 21). For example, some report increased glucose or amino acid transporter expression, whereas others observe no change or downregulation (20). Similar inconsistencies are reported for angiogenic factors, antioxidant defence markers, and inflammatory mediators (22, 23, 24). These discrepancies likely reflect a combination of biological heterogeneity, variation in glycaemic control, gestational age, tissue sampling location, and differences in laboratory platforms and analytical pipelines. Collectively, this inconsistency underscores the need to synthesise evidence across studies to identify patterns of molecular convergence and clarify which placental pathways may be most affected by maternal diabetes.

Given this variability, an integrated evaluation of available high-throughput datasets is required to determine whether reproducible molecular signals emerge despite methodological and biological heterogeneity. Systematic synthesis of omics data can help identify areas of mechanistic convergence and highlight pathways that appear robust across studies. Importantly, because the underlying datasets differ in sample size, analytical thresholds, and technical approaches, this review is explicitly exploratory and intended to generate hypotheses rather than definitive mechanistic conclusions.

High-throughput approaches, such as transcriptomics, proteomics, and metabolomics, are powerful tools for mapping disease-associated pathways and interrogating molecular networks altered in diabetic pregnancy (25). Here, we aimed to identify human placental omics studies comparing uncomplicated and diabetic pregnancies, examine these datasets collectively, and determine the most recurrent molecular changes associated with maternal diabetes. By highlighting convergent pathways, this synthesis provides a foundation for future mechanistic studies and, in the longer term, may inform the development of improved diagnostic or therapeutic strategies.

## Methods

### Search strategy and study selection

The protocol for this systematic scoping review is registered at Research Registry (reviewregistry1882).

A structured search was conducted in PubMed on 16 April 2024, without any filters, using terms related to i) diabetes, ii) placenta, and iii) high-throughput experiments. The full search strategy is provided in ESM Table 1 (see section on Supplementary materials given at the end of the article). Studies were eligible if they included human placental tissue from uncomplicated pregnancies and from pregnancies complicated by diabetes (GDM, T1DM, or T2DM) and reported results from omics analyses. Extensive inclusion and exclusion criteria are listed in ESM Table 2.

**Figure 1:**
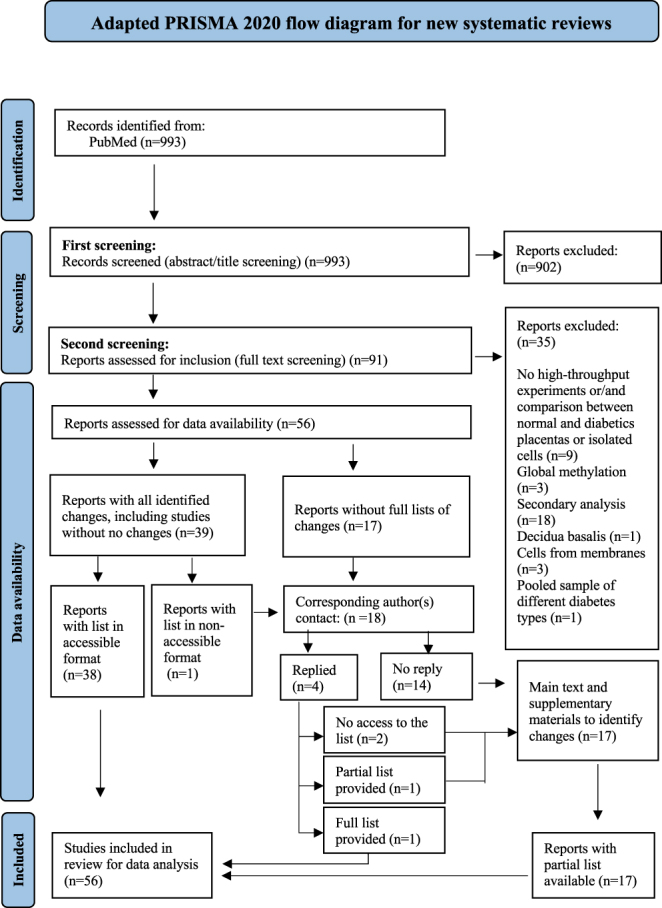
PRISMA flow diagram. Selection process of eligible studies after inclusion and exclusion criteria and data availability assessment.

Screening of identified studies was performed using the Covidence systematic review platform (26). Title and abstract screening and full-text screening was completed by two reviewers. Any discrepancies were resolved through discussion with a third reviewer. The selection process of studies is summarised in PRISMA flow diagram (Fig. 1; ESM Table 3).

Data availability was assessed for all 56 studies. Of these, 38 studies had the complete list of changes available in the main text or supplementary materials. Another 17 studies did not provide a complete list of differentially abundant molecules, and one study presented results in a low-quality visual format. The corresponding authors of these 18 studies were contacted to request the full list, with some providing complete or partial lists. Following this assessment, all 56 studies were retained for inclusion: 39 with full datasets and 17 with incomplete datasets (i.e. only top-ranked or most significant changes available).

### Data extraction

Information was extracted manually using a standardised template capturing: i) study characteristics (title, authors, year of publication, and corresponding author(s) contact information), ii) participant and clinical information (uncomplicated and diabetic placentas, diabetes type, diagnosis criteria, treatment, and gestational age), iii) sample details (placental tissue or isolated placental cells and processing steps, including decidua removal), iv) omics methodology, v) the number and direction of differentially abundant molecules, vi) full lists of altered transcripts and proteins, and vii) criteria used to define statistical significance.

Initial extraction was performed by one reviewer. As part of our internal quality-control workflow, a second reviewer independently cross-checked all extracted variables, and discrepancies were resolved with input from a third reviewer.

### Data analysis and visualisation

Studies were first categorised according to diabetes type (GDM, T1DM, and T2DM) and then stratified by model (whole placental tissue or isolated cells) and omics platform. Only transcriptomic and proteomic datasets were included in the comparative analyses, due to the small number of studies using other platforms. Lists of differentially abundant molecules, as defined by each study’s original statistical thresholds, were used as the basis for downstream analyses. To ensure consistent annotation across all studies, all identifiers were converted to gene symbols using different tools listed in ESM Table 4.

Comparative analyses were performed to identify transcripts or proteins consistently altered in the same direction in placentas from i) GDM, ii) T1DM, and iii) T2DM. Molecules reported by only one study were excluded from convergence analysis. Shared molecules with consistent directionality were submitted to g:Gost in g:Profiler (default settings; significant threshold; g:SCS, 0.05) for functional enrichment analysis (FEA). circRNAs were excluded as they are not reliably represented in gene-based enrichment databases. To account for between-study variability, the full list of differentially abundant molecules from each study was also analysed independently in g:Profiler (27), and recurrently enriched terms were identified.

Similarities between studies were quantified using the Jaccard index for both molecules and enriched terms (28) (https://molbiotools.com/listcompare.php). Protein–protein interaction (PPI) networks were generated using STRING (version 12.0) (29). Network clustering employed *k*-means, with *k* = 15 selected following comparison of *k* = 10, 15, and 20, as *k* = 15 produced stable, interpretable clusters without excessive fragmentation. Data visualisation was performed in R (version 4.3.1) using ggplot2 (30) and corrplot (31).

## Results

### Overview of included studies

**Figure 2:**
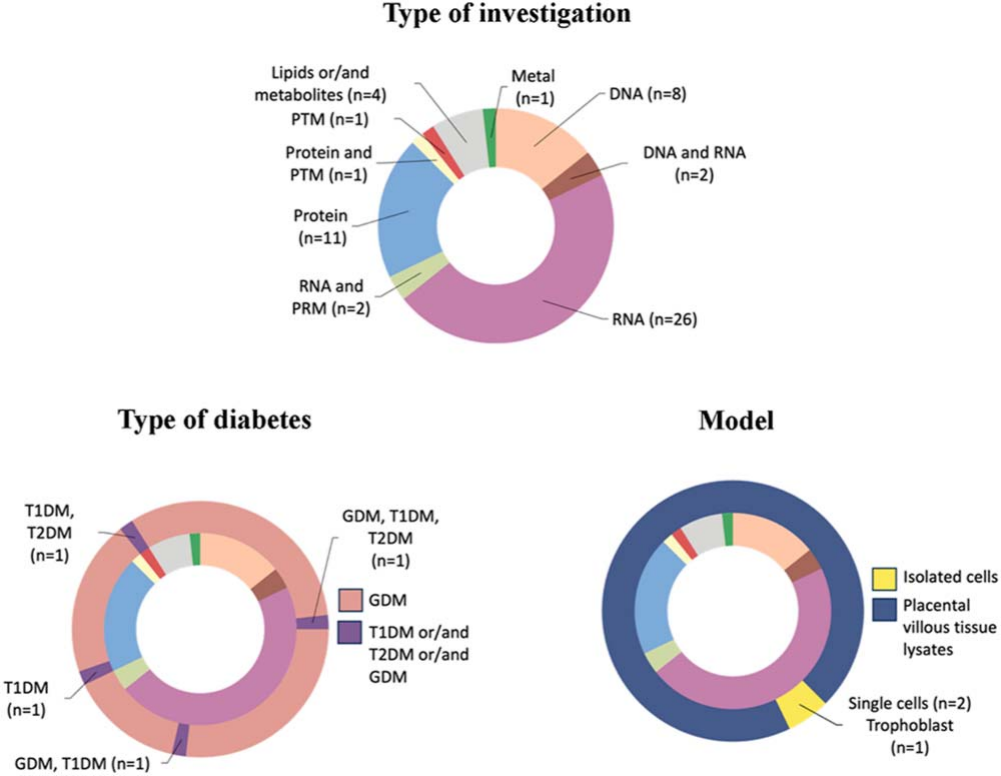
Overview and categorisation of included studies. Categorisation of studies according to type of investigation, type of diabetes, and model. The inner cycle of type of diabetes and model charts represent the main data from the type of investigation chart. The outer circles show additional details related to the studies in the inner circle. PRM, post-transcriptional modification; PTM, post-translational modification; GDM, gestational diabetes mellitus; T1DM, type 1 diabetes mellitus; T2DM, type 2 diabetes mellitus.

A total of 56 studies met our inclusion and exclusion criteria. Four studies investigated molecular changes in placental tissue from pregnancies complicated by T1DM compared with uncomplicated pregnancies (32, 33, 34, 35) (Fig. 2). Two of these also analysed RNA expression in GDM placentas (34) and in T2DM placentas (35), while the other two studies examined protein abundance (32) or post-translational modifications (33), with the latter also including a T2DM cohort (Fig. 2). The remaining 52 focused exclusively on GDM. Of these, one used isolated trophoblast cells (36), two performed single-cell analyses on placental lysates (37, 38), and 49 used villous tissue lysates (Fig. 2). Across the 54 studies that included a GDM comparison, eight investigated DNA methylation changes (39, 40, 41, 42, 43, 44, 45, 46), two assessed both DNA methylation and RNA expression (47, 48), 26 examined RNA expression alone (21, 34, 35, 36, 37, 38, 49, 50, 51, 52, 53, 54, 55, 56, 57, 58, 59, 60, 61, 62, 63, 64, 65, 66, 67, 68), two assessed RNA expression and post-transcriptional modifications (69, 70), ten reported changes in protein abundance (71, 72, 73, 74, 75, 76, 77, 78, 79, 80), one examined in protein abundance and post-translational modifications (81), four assessed lipids and/or metabolites (82, 83, 84, 85), and one investigated metals (86) (Fig. 2). Given that the overwhelming majority of studies examined GDM placentas and that transcriptomic and proteomic datasets were most commonly available, subsequent comparative analyses focused on identifying consistent molecular changes within GDM placentas, with results from T1DM and T2DM included where data permitted.

### Proteomic changes in GDM placentas

Eleven studies examined altered protein abundance in GDM placentas (71, 72, 73, 74, 75, 76, 77, 78, 79, 80, 81) (ESM Table 5). Across these datasets, 15 proteins were reported as differentially abundant in at least two studies; however, only eight demonstrated consistent directionality of change (Table 1). Of these, human chorionic somatomammotropin 1 (CSH1)/placental lactogen was the only protein altered in three studies.

**Table 1:** Consistently identified differentially abundant proteins and RNAs in GDM placentas and their direction of change.

	No. of studies	Relative abundance in GDM placentas	Inter-study agreement in direction of change
15 shared proteins and their regulation			
CSH1	3	Higher	Agreement
CAT, COL14A1, LGALS1, VIM	2	Higher	Agreement
FGA, FGB, MPDZ	2	Lower	Agreement
EPS8L1, HBM, ANXA5, RPL13, SNCA, SPTB, TFRC	2	Higher/lower	Conflict
414 shared RNAs and their regulation			
LIPG, MMP12	4	Higher	Agreement
COL1A1, HSPA1A, LAMA2, LPL, PLA2G5	4	Higher/lower	Conflict
CGB3, KIF26B, THBS1	3	Higher	Agreement
ALDH1A2, DDIT4, EPYC, EVI2A, IGFBP2, IGFBP6, JCHAIN, LAMA3, LUM, NKG7, PRL, SLPI	3	Lower	Agreement
AGTR1, BHLHE40, CHKB, COL17A1, COL5A3, COL6A1, COX4I2, CXCL10, CXCR2, EGFLAM, EMP1, ERRFI1, FAM118A, FLT1, FSTL3, G0S2, GJC1, IL27RA, KCNK17, LAMA5, LEP, MMP9, NDRG1, NES, NOTUM, NR2F1, OLFM4, PDGFRB, PLEKHG2, PLVAP, PTPRB, PTX3, S100A8, SEMA3G, SERPINA1, SLIT3, TBX2, TM4SF1, TMEM74B, WNT3A	3	Higher/lower	Conflict
ACTN1, ADAM12, ADAMTS1, ADGRL1, ANGPT2, ARHGAP42, ATP13A2, BSN, CASP4, CCN2, CCND1, CD93, CDH3, CEBPA, CGB2, CGB5, CGB7, CPXM2, CRISPLD1, DAB2IP, DACH1, DAPK1, DDX11, DEFA1, DGKD, DSCR4, ELMO1, EPHB2, ERC2, FABP4, FAT1, FCGBP, FGF1, FLT4, FRAS1, HCAR1, HSPG2, HUNK, INHBA, ISM2, ITGAX, ITGB8, JAG1, JAG2, KANK3, LAMB1, LAMC3, LARGE2, LDLR, LRP5, MCAM, MCOLN3, MIR-142-3P, MIR503HG, MOCS1, MRC2, MYCN, MYO7A, NCKAP5L, NEB, NELFCD, NOVA2, PCDH17, PDE5A, PLEC, PLEKHA6, PLIN2, PLK1, RCN3, RGS6, SCD5, SEPTIN3, SLC13A3, STAT4, TAC3, TBX4, TCF7L2, TENM3, TIGD1, TLL1, TMEM184B, TNMD, UCK2, VANGL2, VCL, VWCE, WNK2, XIAP, ZDHHC8	2	Higher	Agreement
ACKR1, ACP5, ADGRE2, ADORA3, AHNAK2, ALDOC, ANKRD37, ARHGAP45, ATOH8, BIRC7, C1ORF162, C5AR1, CCDC183, CD2, CD74, circETFA, circPAPPA, circPRKAR1B, circZNF236, CLEC4A, CXCR1, CYTIP, DEFB1, DKK1, DPYSL3, EFNB2, F2R, FOXQ1, GBP5, HK2, HLA-DMA, HLA-DPA1, HLA-DPB1, HLA-DQA1, HLA-DQA2, IGFBP1, IGFBP4, IGHG4, IL15, IL1R2, INHBB, ISG15, KCNK1, KIR2DL4, KLRB1, KRT6C, KRTAP26-1, LGALS1, LNPK, MEDAG, MEFV, MGAM, MGP, MIR-4488, MOXD1, MSLN, NDP, NLGN4X, P4HA1, PCDH20, PENK, PILRA, PLA2G4A, PPP1R1C, PRUNE2, PWWP3B, RBP1, SCGB1D2, SCNN1A, SIK1, SLAMF7, SLC11A1, SLC16A6, SLC2A5, SRPX2, SULF2, SYTL3, TAFA4, TFRC, TLE6, TLR2, UPK1B, WWC3	2	Lower	Agreement
ABCA7, ACE, ACSL1, ACSL3, ADAMTS18, ADCY4, ADGRA2, ADGRF5, ADRB3, AEBP1, AFAP1L2, AGPAT5, ALAS2, APOLD1, ATP12A, ATP1B2, BMP2, BMP4, BMP5, BTBD16, CALD1, CCDC102B, CCDC3, CCDC68, CCL2, CCN3, CD52, CD63, circEXOC6B, circNCOR1, circPHC3, circPTBP3, circRNF111, circZFAT, CLK1, COL14A1, COL15A1, COL1A2, COL5A1, COL6A3, COLEC10, CSPG4, CST7, CTSS, CXCL1, CYP19A1, DIRAS1, DNAJC3, DOCK5, DSC1, DSG3, EBF1, EGFR, ELN, EPHA3, FAM162B, FBLN1, FCHSD1, FCN3, FN1, FOSL2, FOXS1, FZD1, GALNS, GBF1, GBP1, GDF15, GJA4, GKN1, GLT8D2, GREB1L, GUCY1A1, HAPLN1, HAPLN3, HAS2, HBE1, HBG1, HEY2, HEYL, HIF3A, HIGD1B, HLA-B, HLA-DRA, HLA-G, HOXA13, HSPH1, IFI30, IL1RL1, IL2RB, IQSEC1, ITGA9, KCNK12, KCNS3, KLHL3, KRT17, KRT5, KRT6A, LCN2, LDB3, LINC00474, LINGO1, LPAR6, LRP6, LRRC45, LRRC4B, LRRN3, LTBP2, LYVE1, MCEMP1, MECOM, MEOX2, MIDEAS, MIF, MMP1, MMP14, MMP2, MSRB3, MUC20, NEURL2, NID1, NOTCH3, NRIP1, OLAH, OLFM2, OLFML1, OR51E1, OR51E2, PCDH18, PCSK6, PDE3A, PDXK, PLAAT3, PLAC9, PLAGL1, PORCN, PPP2R2C, PREX2, PTPRD, RAC3, RAPGEF5, RASL12, RDH13, RGL2, RMRP, RNASE4, RNF180, RSRP1, SASH1, SCD, SCUBE2, SERPINB7, SFRP1, SGSM1, SH3BP5, SH3PXD2B, SIGLEC6, SLC34A2, SLCO4A1, STARD13, STEAP4, STMN1, SYDE1, SYT8, TBX5, TKT, TM4SF18, TMEM100, TNS2, TOB2P1, TOX3, TRGC1, TRMT9B, TRPC6, TSPAN14, UNC5B, VEGFA, VIT, WDR91, XDH, ZEB2	2	Higher/lower	Conflict

### Transcriptomic changes in GDM placentas

Twenty-seven studies assessed RNA expression in GDM placentas using microarray (*n* = 9), RNA sequencing (*n* = 17), or the GeneCalling method (*n* = 1) (87) (ESM Table 6). Most studies focused on mRNA and/or non-coding RNA. A subset analysed circRNAs exclusively (*n* = 3) (49, 64, 66), miRNA (*n* = 5) (53, 56, 57, 65, 67), or long non-coding RNA (lncRNA) (*n* = 1) (52), while one study reported circRNA, miRNA, lncRNA, and mRNA together (63). Three studies detected no significant differences (35, 53, 55), leaving 24 studies for comparative analysis.

Across these 24 studies, 414 RNAs were reported as differentially abundant in at least two datasets (Table 1). Among these, 189 showed consistent directionality: 94 upregulated (mRNA: 91, miRNA: 1, and lncRNA: 2) and 95 downregulated (mRNA: 90, circRNA: 4, and miRNA: 1) in GDM compared to uncomplicated placentas (Table 1). Endothelial lipase (*LIPG*) and matrix metallopeptidase 12 (*MMP12*) were upregulated in four studies, while chorionic gonadotropin subunit beta 3 (*CGB3*), kinesin family member 26B (*KIF26B)*, and thrombospondin 1 (*THBS1*) were upregulated across three (Table 1).

### Integrated RNA and protein signatures in GDM placentas

Across GDM studies, 98 genes were altered at both the RNA and protein levels (Table 2). Forty-seven of these showed consistent directionality, representing a subset of molecules with reproducible dysregulation across platforms (Table 2).

**Table 2:** Consistent and divergent RNA–protein regulation across studies in GDM placentas.

98 shared proteins and corresponding mRNAs and their regulation	No. of studies		Abundance in GDM placentas between RNA and protein studies		Agreement between RNA and protein
	RNA	Protein	RNA	Protein	
FLT1, HSPA1A, LPL	4	1	Higher/lower	Lower	Conflict
SERPINA1, TMEM184B	3	1	Higher/lower	Lower	Conflict
COL5A3, NES	3	1	Higher/lower	Higher	Conflict
COL14A1	2	2	Higher/lower	Higher	Conflict
TFRC	2	2	Lower	Higher/lower	Conflict
LGALS1	2	2	Lower	Higher	Opposite direction
DPYSL3	2	1	Lower	Lower	Agreement
DAPK1, TAC3	2	1	Higher	Lower	Opposite direction
RDH13, IQSEC1, HBG1, COL15A1	2	1	Higher/lower	Lower	Conflict
NID1, PDXK, AFAP1L2, CALD1	2	1	Higher/lower	Higher	Conflict
HSPG2, LAMC3, MOCS1	2	1	Higher	Higher	Agreement
HBM	1	2	Lower	Higher/lower	Conflict
EPS8L1	1	2	Higher	Higher/lower	Conflict
ANXA4, ARL2BP, BRD4, BTF3, CD34, CSH2, EPB41L1, EXPH5, FLNA, GH1, LDHB, MYL9, P4HB, PCTP, PEAR1, POR, RAB5C, SLC25A10, TACC2, TGFB1I1, VWF	1	1	Higher	Higher	Agreement
ANK1, APOA1, BCLAF1, C1QTNF6, C7, CD109, CD248, CLIC2, CLU, CRH, DSC3, EMILIN2, GLYR1, GPX3, GRAMD4, HLA-A, HSPA1B, ISLR, SERPINF2, SLC4A1, TAGLN2, TXN	1	1	Lower	Lower	Agreement
ACTA2, ASPA, B3GNT3, LTBP3, MFAP5, MICAL1, PKP3, THSD7A, GRAMD1B, KRT1, SLCO2A1, SRRM1, TAGLN	1	1	Higher	Lower	Opposite direction
A2M, AKAP12, C4A, COL6A2, DDX5, ENPP1, F13A1, GNAI1, GNAQ, GPX7, HADHA, MYH11, NNMT, RSPO3, S100A6, UBQLN1	1	1	Lower	Higher	Opposite direction

**Table 3:** Differentially abundant RNA in type 1 and type 2 diabetes mellitus compared to uncomplicated placentas. Bold indicates changes in gene expression in GDM compared to uncomplicated placentas, while underline indicates the same directional changes between GDM and type 1 or 2 diabetes mellitus.

Diabetes type	Regulation
Type 1 diabetes mellitus	
***ACSL3****, AMACR,* ***APOE****, B4GALT1, B4GALT7,* ***CEBP****, EBP,* ***FABP4****,* ***FABP5****, FUT5,* ***HADH****, HYAL4,* ***LIPG****,* ***LRP1****, MAN1A1,* ***MGAT1****, MGAT2,* ***NR2F2****, OGT,* ***OLR1****,* ***PLTP****,* ***SLC2A10****,* ***ST3GAL1****, TGM2*	Upregulated
***GNE****,* ***LPL***	Downregulated
Type 2 diabetes mellitus	
*ACSL6,* ***ADD2****,* ***AHSP****,* ***ALAS2****,* ***ANK1****,* ***APOLD1****, ARID5A, BBOF1,* ***BMP2****, C17ORF99,* ***C5AR1****, CNNM2, CYTH1, DCAF12, EEF1A1P13,* ***EPB42****,* ***FAM210B****, FECH, FILIP1L, FOXO3, GALNT5, GCAWKR,* ***GEM****, GFI1B, GMPR, GYPA,* ***GYPB****, GYPE, HBA1, HBA2, HBEGF,* ***HBG2****,* ***HBM****,* ***HEMGN****, HMBS,* ***IGSF9B****,* ***KLF9****,* ***KRT24****, LBH,* ***MIR-758****,* ***MYL4****,* ***NNMT****, OSBP2, PALM2AKAP2, PIK3CD, PLAUR, QPCTL, R3HDM4, RELB, RHAG, RHCE, RHD, RIPOR3, RYR3,* ***SAA1****,* ***SCN3A****, SELENBP1,* ***SIK2****, SLC17A4, SLC25A37,* ***SLC25A39****, SLC38A11, SLC45A1,* ***SLC4A1****, SLC6A1,* ***SOCS3****,* ***SOX9****,* ***SPTA1****, TFR2,* ***THBS1****,* ***THEMIS2****, TRIM10,* ***XK****,* ***YOD1****, ZHX3*	Upregulated
*ENSG00000273035, ESCO1, GATAD1, IKZF2, KLHDC2, MYCBP2, PSG5, RMI1, SCYL3, SFPQ, SMARCA5, SRGAP2C, SSBP3-AS1, TIGD2, TMEM69,* ***TRIM33****,* ***TRPM7****, USP25*	Downregulated

### Functional enrichment and network analysis (FEA)

**Figure 3:**
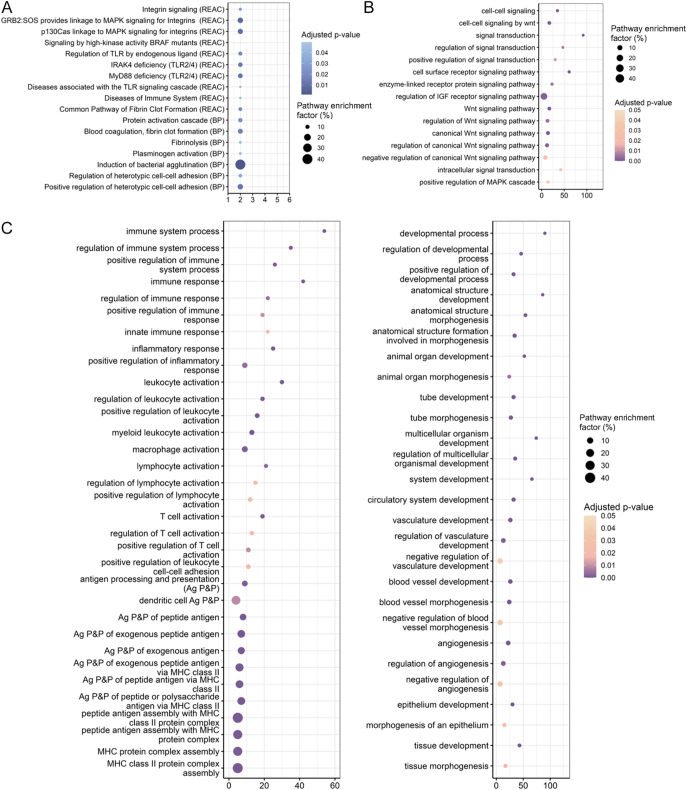
Functional enrichment analysis of differentially abundant proteins and RNAs across GDM studies. FEA of 8 proteins (A) and 189 RNAs (B and C) with consistent directionality changes across studies ((B) terms related to signalling; (C) terms related to immunity and vasculature). The size of bubbles represents the pathway enrichment factor (percentage of intersection size divided by term size), with larger bubbles indicating a higher enrichment factor. The x-axis reflects the number of genes (representing proteins/RNAs) in the query annotated to each term. The bubble colour indicates adjusted *P*-value, with darker colour representing a lower adjusted *P*-value.

**Figure 4:**
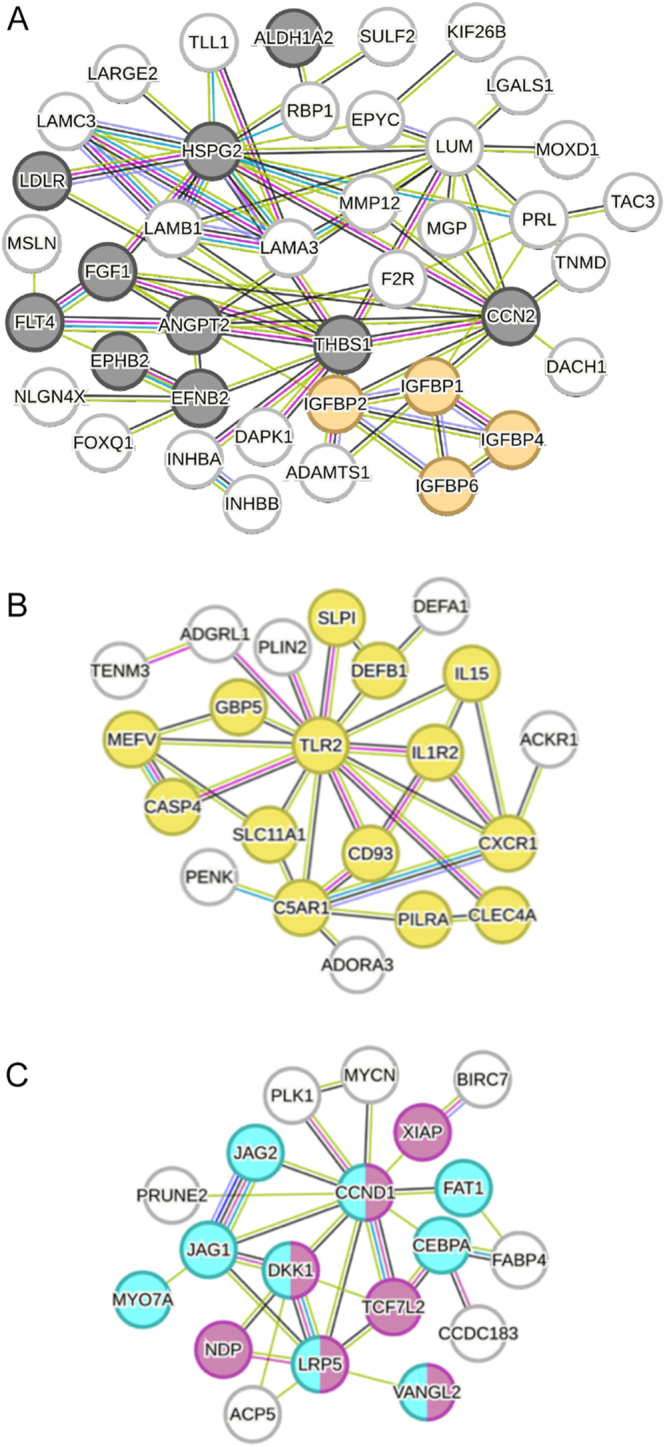
The functional modules of 189 RNAs highlight biological process networks. (A, B, C) Clustering of the network of 189 RNAs using k-means in STRING identified 15 distinct clusters with the three largest being associated with (A) blood vessel development (black), regulation of insulin-like growth factor receptor signalling pathway (orange), (B) immune system (yellow), (C) epithelium development (light blue), Wnt signalling pathway (pink).

FEA of consistently altered molecules identified processes related to Wnt signalling, immune regulation, vascular development, and metabolic responses (Fig. 3B and C; ESM Fig. 1). At the protein level (*n* = 8), altered molecules associated with the FEA terms included fibrinogen chains FGA and FGB (Fig. 3A). Network analysis of the 189 commonly altered genes yielded a PPI network containing 180 nodes and 288 edges, which clustered into 15 modules. The largest cluster was enriched for angiogenesis and IGF signalling, with central nodes including heparan sulphate proteoglycan 2 (HSPG2), thrombospondin 2 *(THBS2)*, lumican (*LUM*), CCN family member 2 (*CCN2*), angiopoietin-2 (*ANGPT2*), fibroblast growth factor 1 (*FGF1*), and insulin-like growth factor-binding protein 2 (*IGFBP2*) (Fig. 4A). A second major cluster contained immune-related genes (Toll-like receptor 2 (*TLR2*), C5a anaphylatoxin chemotactic receptor 1 (*C5AR1*), C-X-C chemokine receptor type 1 (*CXCR1*), and interleukin-1 receptor type 2 (*IL1R2*); Fig. 4B). A third cluster reflected Wnt/epithelial development signalling, with cyclin D1 (*CCND1*) as a key node connected to low-density lipoprotein receptor-related protein 5 (*LRP5*), dickkopf-related protein 1 (*DKK1*), jagged-1 (*JAG1*), and CCAAT/enhancer-binding protein alpha (*CEBPA*) (Fig. 4C). Molecules altered at both RNA and protein levels (*n* = 47) were enriched for coagulation, platelet activity, and wound-healing pathways (ESM Table 7).

### Cross-study functional enrichment analysis

**Figure 5:**
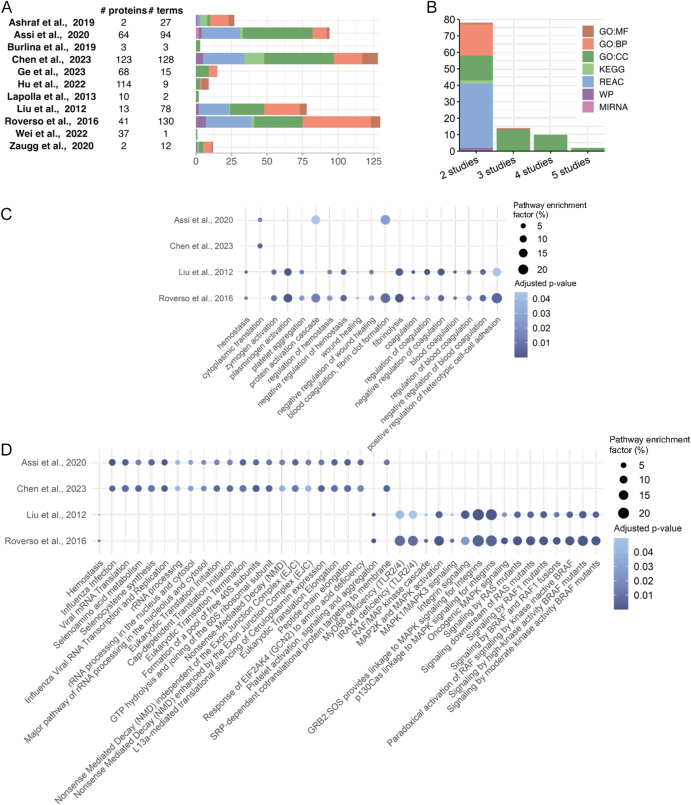
Cross-study GO and pathway enrichment of differentially abundant placental proteins in GDM. (A) Number of terms identified (x-axis) using the available list of protein changes for each study and (B) shared terms between at least two studies (colour indicate different FEA categories). (C) Biological process and (D) Reactome pathways that are enriched between studies. The y-axis shows the study in which each GO and FEA term (x-axis) was identified. The bubble size represents the pathway enrichment factor (percentage of intersection size divided by term size), with larger bubbles indicating a higher enrichment factor and colour indicating adjusted *P*-value, with darker colour representing a lower *P*-value. GO, gene ontology; MF, molecular function; BP, biological process; CC, cellular component; KEGG, Kyoto Encyclopedia of Genes and Genomes; REAC, Reactome; WP, WikiPathways.

To enhance sensitivity to biologically meaningful convergence, FEA was performed within each study and shared terms were compared across datasets (88). This approach increased inter-study similarity (ESM Fig. 2A and B) compared with direct overlap of individual molecules (ESM Fig. 2C and D); however, it should be interpreted with caution as variability may reflect biological differences between studies, but it may also arise from differences in experimental design, sample size, and bioinformatics pipelines. Across proteomic studies, 104 biological processes or pathways were shared by at least two datasets (Fig. 5A and B). These included haemostasis, fibrinolysis, coagulation (Fig. 5C), RNA processing, immune activation cellular stress responses, platelet function, selenoamino acid metabolism, and MAPK signalling (Fig. 5D).

**Figure 6:**
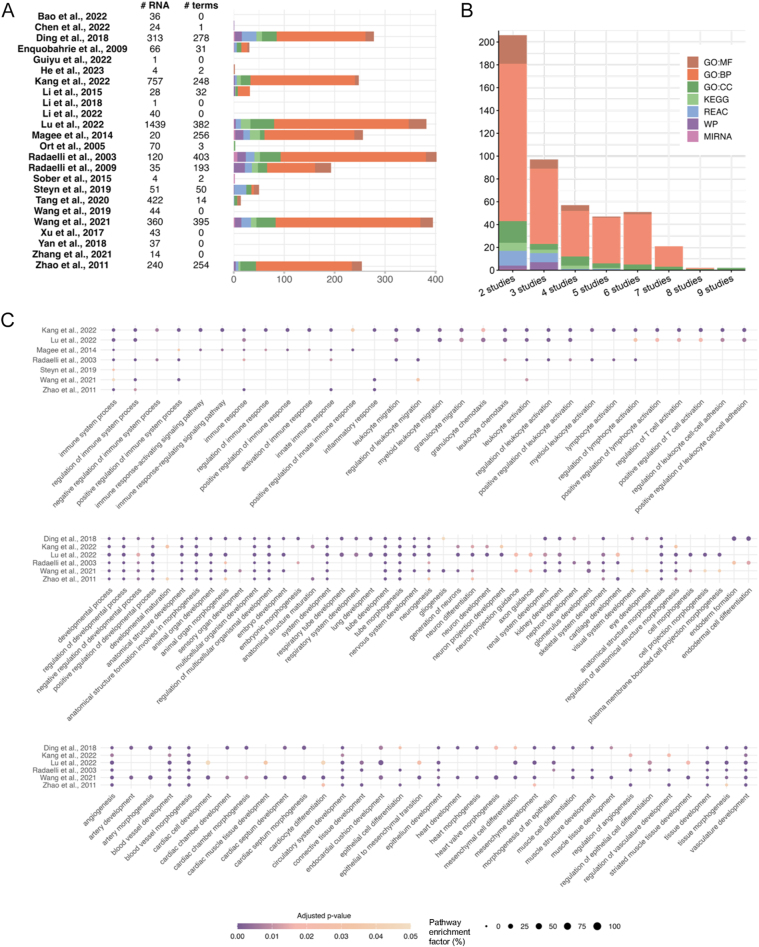
Cross-study GO and pathway enrichment of differentially abundant placental genes in GDM. (A) Number of terms identified (x-axis) using the available list of RNA changes for each study and (B) shared terms between at least two studies (colours indicate different GO and FEA categories). (C) Biological process related to immune system, development, and vascular system are enriched between studies. The y-axis shows the study in which each GO and FEA term (x-axis) was identified. The bubble size represents the pathway enrichment factor (percentage of intersection size divided by term size), with larger bubbles indicating a higher enrichment factor and colour indicating adjusted *P*-value, with darker colour representing a lower *P*-value. GO, gene ontology; MF, molecular function; BP, biological process; CC, cellular component; KEGG, Kyoto Encyclopedia of Genes and Genomes; REAC, Reactome; WP, WikiPathways.

**Figure 7:**
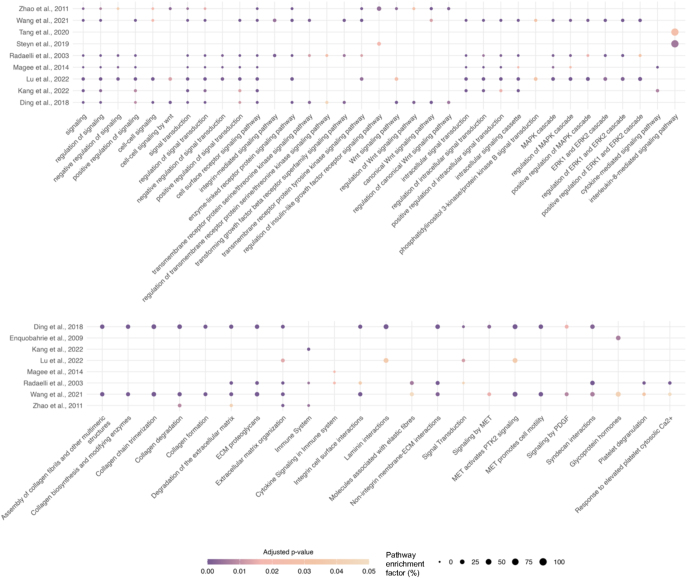
Shared signalling and Reactome pathways across transcriptomic studies in GDM placentas. The y-axis shows the study in which each GO and FEA term (x-axis) was identified. The bubble size represents the pathway enrichment factor (percentage of intersection size divided by term size), with larger bubbles indicating a higher enrichment factor and colour indicating adjusted *P*-value, with darker colour representing a lower *P*-value.

Across the 24 transcriptomic studies (Fig. 6A), 370 terms were shared between at least two studies. Of these, 74 were shared by three studies, 41 by four or five studies, 44 by six studies, and 18 by seven studies. Only one term – ‘response to organic substance’ – was shared by eight studies (Fig. 6B). The most consistently enriched pathways involved immune function, vascular and cardiac development (Fig. 6C), and growth-related signalling pathways, including phosphatidylinositol 3-kinase (PI3K)/Akt, integrins, IGF, TGF-β, Wnt, and MAPK/extracellular signal-regulated kinase (ERK)-1/ERK-2 cascades (Fig. 7). Other enriched terms reflected cellular and multicellular processes, metabolism, and broader response to stimulus (ESM Fig. 3).

### Comparative analysis of molecular changes in GDM, T1DM, and T2DM

Although limited, available T1DM and T2DM studies provided additional context. No high-throughput studies investigating placental proteome were available for T2DM placentas. One study assessed protein expression in T1DM using a 60-protein cell-cycle array in first-trimester placentas (following elective pregnancy termination), identifying four less abundant proteins (MKI67, CHEK1, TP73, and CDC34) compared to uncomplicated placentas (32).

Transcriptomic data were also sparse. Radaelli *et al.* (34) identified 26 differentially abundant RNAs enriched in lipid transport, metabolism, vascular pathways, and protein glycosylation (ESM Fig. 4A), while Kedziora *et al.* (35) reported 93 altered genes enriched in erythrocytes oxygen/carbon dioxide exchange and haematopoiesis (ESM Fig. 4B).

### Comparisons of altered molecules across diabetes types

**Figure 8:**
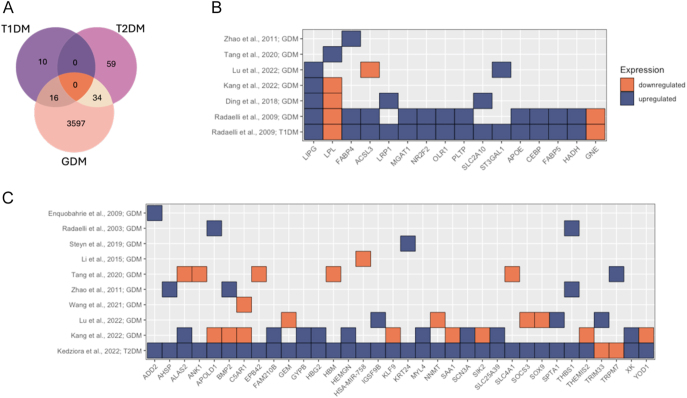
Comparative transcriptomic signatures across GDM, T1DM, and T2DM placentas. (A) Venn diagram showing all genes that are differentially expressed in studies investigating type 1 and type 2 diabetes mellitus and GDM placentas compared to uncomplicated placentas. (B) A total of 16 differentially abundant RNAs were identified in studies examining RNA changes in GDM compared to uncomplicated pregnancies, as well as in a study that investigated changes in type 1 diabetes mellitus relative to uncomplicated pregnancies. (C) A total of 34 differentially abundant RNAs were reported in studies analysing RNA changes in GDM compared to uncomplicated pregnancies, along with a study that assessed gene expression changes in type 2 diabetes mellitus compared to uncomplicated pregnancies. (B and C) The y-axis represents the studies in which gene expression changes were observed, with the final study focusing on pre-existing diabetes. The x-axis displays the genes with expression changes in each study, where blue indicates upregulation and orange indicates downregulation (the grey background represents genes that were not identified as differentially abundant in the list used from the respective study). GDM, gestational diabetes mellitus; T1DM, type 1 diabetes mellitus; and T2DM, type 2 diabetes mellitus.

Due to the absence of third-trimester T1DM and T2DM proteomic data sets, comparisons across diabetes types were restricted to transcriptomic studies (Fig. 8A). Of the 26 genes altered in T1DM (Table 3), 16 overlapped with GDM, 14 of which showed consistent directionality and were associated with lipid handling, vascular processes, and adipogenesis (Fig. 8B; ESM Fig. 4C). Of the 93 RNAs altered in T2DM (Table 3), 34 overlapped with GDM (Fig. 8C), although only 14 showed consistent directionality and were not associated with enriched pathways. No molecules were consistently altered across all three diabetes types.

## Discussion

This systematic review consolidated evidence from high-throughput studies of placentas from diabetic pregnancies and identified recurrent transcriptomic and proteomic alterations despite substantial heterogeneity across individual reports. Data on T1DM and T2DM remain sparse, but across GDM studies, although inter-study differences exist, we found eight proteins and 189 RNAs consistently altered in the same direction in at least two independent studies, with the most reproducible signals enriched in immune and vascular pathways. To account for variation in analytical methods and significance thresholds across studies, we additionally performed FEA within each dataset and compared shared terms (88, 89). This strategy, although rarely applied in placental research, increased inter-study convergence and highlighted biological pathways that may be robust to methodological differences. Experimental validation will be required to determine if these pathways are casual rather than correlative. Across analyses, several pathways central to placental growth and adaptation appeared repeatedly, including integrin, PI3K/Akt, IGFBP, TGF-β, Wnt, and MAPK/ERK signalling. These pathways regulate trophoblast turnover, angiogenesis, nutrient transport, and cellular metabolism, and their recurrent enrichment supports the view that maternal hyperglycaemia disrupts core regulatory networks rather than isolated genes.

Among these pathways, the IGF–IGFBP axis emerged as a consistent theme. Multiple studies report reduced IGFBP expression in GDM placentas (90), including IGFBP1 (49, 61), IGFBP2 (47, 61, 67), IGFBP4 (47, 67), and IGFBP6 (47, 61, 67). Although some IGFBPs perform IGF-independent roles (91), their primary function at the maternal–fetal interface is to regulate IGF bioavailability. IGF-I and IGF-II are key regulators of human placental development and nutrient transport via the type 1 (IGF1R) and downstream MAPK and PI3K/Akt pathways. Increased IGF1R activation has been reported in hyperglycaemic pregnancies, suggesting that altered IGF signalling may contribute to excessive nutrient transfer, placental overgrowth, and abnormal vascular development in GDM. Reduced placental and circulating IGFBP1 have also been linked to insulin resistance (92, 93, 94), while IGFBP1 phosphorylation status may influence fetal growth (93, 95). Collectively, this supports dysregulation of the IGF–IGFBP axis as a potential mediator of GDM-related placental dysfunction. However, mechanistic conclusions cannot be drawn from the available high-throughput studies and should be explored experimentally.

Vascular pathways were also among the most consistently enriched across transcriptomic and proteomic datasets. This aligns with reports of impaired placental vascular development in diabetes (96, 97). Components of the IGF axis may contribute to these vascular alterations, given evidence from animal models that IGF2R–ERK1/ERK2 signalling regulates placental microvasculature (98, 99), although equivalent mechanisms in humans remain unclear. TGF-β signalling also warrants consideration, as it regulates endothelial-to-mesenchymal transition (EndMT) (100). Our previous work demonstrated altered TGF-β signalling and evidence of EndMT in GDM placentas (101), and one of the commonly altered lncRNA in this review, MIR503HG, regulates both TGF-β signalling (102) and EndMT (103). These findings raise the possibility that lncRNA-mediated modulation of TGF-β pathways may contribute to the vascular immaturity that is commonly observed in GDM placenta (24), but further studies are required.

In addition to vascular changes, inflammatory and immune-related pathways were strongly represented. This is notable given evidence linking GDM to sterile inflammation in the placenta (104, 105) and increased risk of stillbirth and neonatal morbidity (106, 107). Hofbauer cells, the principal immune cells in the chorionic villi of the placenta (108), are altered in GDM and may contribute to these signatures (109). Immune dysregulation may represent key driver of altered placental function, although the specific initiating signals remain poorly defined; studies in *ex vivo* human placental explants suggest that they could be driven directly by maternal hyperglycaemia (110).

Importantly, however, placental abnormalities persist (111), even in pregnancies with well-controlled glycaemia (24, 112), indicating that maternal glucose alone does not explain all of the observed molecular changes. GDM is a systemic condition involving alterations in insulin, adipokines, lipids, and extracellular vesicles (EVs) (113, 114, 115), all of which could influence placental signalling. Few studies have directly examined how these maternal factors contribute to placental gene regulation, and this represents a key area for future research. Likewise, the impact of different therapeutic strategies (e.g. diet, metformin, and insulin) on placental molecular profiles in diabetes is largely unknown; only one study in this review directly compared treatment groups in GDM (59). As the use of pharmacological interventions increases globally, understanding how treatments modulate placental biology will be essential. Finally, the incidence of T1DM (116) and, particularly, T2DM (117) in women of reproductive age increases, showcasing the urgency of defining how distinct diabetes phenotypes affect placental function. Given the sparse data available for these conditions, further studies are required to characterise their molecular signatures and to determine whether the pathways identified in GDM generalise across diabetes subtypes (118, 119).

### Limitations

Data availability varied substantially across studies, and raw datasets were generally inaccessible, preventing re-analysis using a unified bioinformatic pipeline. As a result, we relied on authors’ reported thresholds for differential abundance, which differed across studies and likely contributed to variability in the number and type of molecules identified. Incomplete reporting of non-significant molecules also made it difficult to distinguish between true absence of effect and lack of detection. Technical factor difference in sequencing depth, protein detection limits, library preparation, and use of different genome assemblies introduced further variability, as did inconsistency in placental sampling sites and processing methods. Comparability across diabetes types was limited, particularly because the only T1DM proteomic dataset was derived from first-trimester tissue, whereas GDM data sets were from term placentas. Clinical and demographic variables were also inconsistently reported, restricting assessment of key confounding influences, such as BMI, fetal sex, glycaemic control, medication use, and comorbidities. In addition, most studies had small samples sizes, reducing power and stability of identified signatures. Finally, as this work adopts an exploratory approach synthesising available evidence to identify emerging patterns, it should be interpreted as preliminary and hypothesis-generating, requiring targeted validation in future studies.

## Conclusion

This synthesis identifies recurring transcriptomic and proteomic alterations in GDM, highlighting dysregulation of pathways controlling growth, vascular development, and immune signalling. These patterns provide insights into how maternal diabetes may disrupt placental biology and suggest pathways for further mechanistic investigation. Future studies should incorporate larger, better characterised cohorts, harmonised analytical pipelines, and systematic evaluation of effects, as well as dedicated analyses of T1DM and T2DM to improve the understanding of how different diabetes states influence placental gene regulation and pregnancy outcomes.

## Supplementary materials



## Declaration of interest

EMS received honoraria for talks and workshops from Abbott Diabetes Care, Lilly Diabetes Care, and Ypsomed Diabetes Care. GF, ARB, BH, and KF declare no competing interests related to this work.

## Funding

GF was funded by a Leeds Doctoral Scholarship from University of Leeds. This work was supported by grants from UK Research and Innovation Medical Research Council (MR/R023166/1 and MR/Y003659/1).

## Author contribution statement

GF, BH, and KF conceived the review. GF led the screening of the papers, with ARB as the second reviewer and BH resolving any conflicts that arose. EMS, BH, and KF supervised the study. GF conducted all analyses and visualised the datasets. GF, BH, and KF interpreted the results and drafted the main manuscript. All authors reviewed and approved the final version and have no competitive interest to declare.

## Data availability

Data that have been used or analysed in this review are publicly available or can be received upon contacting the corresponding authors. Any generated data are available in the manuscript.
