# Supplementary material for: Decoding the molecular landscape of the placenta in maternal diabetes: a systematic review of high-throughput data
Source: J Mol Endocrinol. 2026 Apr 10;76(3):e250131. doi: 10.1530/JME-25-0131 (PMC13097123; doi:10.1530/JME-25-0131)
Supplement: Supplementary file 1 [file supplementary_materials.pdf]

ESM Table 1: Search strategy

	Search term
1	placenta OR placental OR placentas OR placenta*
2	diabetes OR GDM OR gestational diabetes mellitus OR A1GDM OR A2GDM OR GDMA1 OR GDMA2 OR class A1 gestational diabetes mellitus OR class A2 gestational diabetes mellitus OR diabetic OR diabetic* OR type 1 diabetes OR type 2 diabetes OR T1D OR T2D OR T1DM OR T2DM
3	genomics OR epigenomics OR transcriptomics OR proteomics OR lipidomics OR metabolomics OR glycomics OR phosphoproteomics OR metagenomics OR omic* OR microarray OR array OR sequencing OR high-throughput OR RNA-seq OR single-cell OR single nuclei OR single cell RNA OR GEO OR differentially expressed OR differential expression OR differential abundance OR methylome OR methylation OR glycoproteomics OR metalloproteomics OR fluxomics OR inomics OR integromics OR phenomics OR redomics OR epitranscriptomics OR epi-transcriptomics OR epiproteomics OR epi-proteomics OR metatranscriptomics OR meta-transcriptomics OR metaproteomics OR meta-proteomics OR metagenomics OR meta-genomics OR genomic OR epigenomic OR transcriptomic OR proteomic OR lipidomic OR metabolomic OR metabonomic OR glycomic OR phosphoproteomic OR glycoproteomic OR metalloproteomic OR fluxomic OR inomic OR integromic OR phenomic OR redomic OR epitranscriptomic OR epi-transcriptomic OR epiproteomic OR epi-proteomic OR metatranscriptomic OR meta-transcriptomic OR metaproteomic OR meta-proteomic OR metagenomic OR meta-genomic OR genome OR epigenome OR transcriptome OR proteome OR lipidome OR metabolome OR metabonome OR glycome OR phosphoproteome OR glycoproteome OR metalloproteome OR fluxome OR inome OR integrome OR phenome OR redome OR epitranscriptome OR epi-transcriptome OR epiproteome OR epi-proteome OR metatranscriptome OR meta-transcriptome OR metaproteome OR meta-proteome OR metagenome OR meta-genome OR phosphorylome OR methylome OR acetylome OR ubiquitinome OR omics OR omic OR *omics* OR *omic* OR *omic OR *omics OR high throughput OR sequencer OR RNA sequencing OR RNA-sequencing OR RNAseq OR scRNA-seq OR scRNAseq OR scRNA OR single-cell RNA

	OR sc-RNA-seq OR single-cell OR sequencing OR single-cell RNA-sequencing OR snRNA-seq OR snRNAseq OR snRNA-sequencing OR snRNAsequencing OR single-nucleus OR single-nuclei OR single nuclei OR single nuclei RNA sequencing OR single nuclei RNA-sequencing OR single nuclei RNA seq OR single nuclei RNA-seq OR single nuclei RNAseq OR single-nuclei RNA sequencing OR single-nuclei RNA-sequencing OR single-nuclei RNA seq OR single-nuclei RNA-seq OR single-nuclei RNAseq OR single nucleus RNA sequencing OR single nucleus RNA-sequencing OR single nucleus RNA seq OR single nucleus RNA-seq OR single nucleus RNAseq OR single-nucleus RNA sequencing OR single-nucleus RNA-sequencing OR single-nucleus RNA seq OR single-nucleus RNA-seq OR single-nucleus RNAseq OR Gene Expression Omnibus OR Github OR Pride OR Proteomexchange OR ArrayExpress OR differentially abundant OR differentially methylated OR differential methylation
**Final search strategy**	1 AND 2 AND 3

ESM Table 2: Inclusion and exclusion criteria

**Inclusion criteria**
1. Use of human placental tissue or primary isolated placental cells from uncomplicated and diabetic pregnancies (T1DM, T2DM, or GDM) that performed high-throughput approaches comparing changes in uncomplicated and diabetic samples.
2. Studies that re-analysed publicly available data were eligible for inclusion only if the primary study had not performed analyses comparing diabetic with normal samples.
**Exclusion criteria**
1. Reviews, perspective articles, pre-prints, and studies not available in English were excluded.
2. Also, studies that only used decidual basalis, maternal blood, and tissue or cells isolated from umbilical cord were excluded.
3. Other reasons for exclusion were studies examining the placental microbiome, and studies that only performed experiments in animal models of diabetes, isolated cells from placental membranes, cultured human placental explants, or cell lines.
**Following initial screening utilising the above criteria, further inclusion and exclusion criteria were applied during the full-text screening to identify suitable studies to be used for data analysis**
1. Studies were only included if the list of changes (incomplete or full list) for individual gene/protein/lipid/metabolites etc was available in the main text or supplementary materials or was provided after contacting the corresponding author(s).
2. Studies investigating other pathologies where some patients had diabetes, but that did not perform an analysis comparing diabetics with uncomplicated controls were excluded. Additionally, studies that used high-throughput approaches but only reported global changes (i.e., global methylation levels, total lipid level, etc.) were excluded as the focus of this study was to identify specific common changes and altered pathways between studies. Studies that combined samples from more than one type of diabetes were excluded.
3. Studies that performed data analysis focusing on the relationship of genes without reporting specific genes that were changed between uncomplicated and diabetic placentas (i.e., coexpression network analysis) were excluded.

ESM Table 3: Studies excluded following full text screening

**No high-throughput experiments or/and comparison between normal and diabetics placentas or isolated cells (n=9)**
DOI: 10.1016/j.placenta.2022.11.006, DOI: 10.2337/db19-0798, DOI: 10.21203/rs.3.rs-3464151/v1, DOI: 10.18632/aging.102049, DOI: 10.1111/jog.14820, DOI: 10.1016/j.repbio.2021.100566, DOI: 10.1186/s12884-018-2066-9, DOI: 10.1371/journal.pone.0049248, DOI: 10.1016/j.diabres.2021.109046
**Global methylation (n=3)**
DOI: 10.1007/s11033-023-09005-z, DOI: 10.1177/1933719113492206, DOI: 10.1186/s13148-016-0247-9
**Secondary analysis (n=18)**
DOI: 10.2147/JIR.S440826, DOI: 10.1002/jcb.29584, DOI: 10.1186/s12884-022-04716-w, DOI: 10.1080/21655979.2021.1950279, DOI: 10.17179/excli2017-491, DOI: 10.1210/en.2016-1922, DOI: 10.1177/15353702231199077, DOI: 10.1080/02648725.2023.2215966, DOI: 10.1007/s10528-024-10769-7, DOI: 10.1080/09513590.2020.1712696, PMID: 34149999, DOI: 10.1186/s13148-019-0692-3, DOI: 10.1186/s12958-019-0546-z, DOI: 10.1016/j.arcmed.2023.102925, DOI: 10.1038/s41598-018-34292-z, DOI: 10.1002/mgg3.515, DOI: 10.3389/fendo.2023.1177547, DOI: 10.1007/s11845-021-02838-2
**Decidua basalis (n=1)**
DOI: 10.1186/s13148-015-0116-y
**Cells from membranes (n=3)**
DOI: 10.1007/s00125-018-4699-7, DOI: 10.1186/s13287-020-01828-y, DOI: 10.1042/CS20180825
**Pooled sample of different diabetes types (n=1)**
DOI: 10.1371/journal.pone.0190698

ESM Table 4: Annotation conversion

**Annotation conversion for most genes**
g:Convert in g:Profiler (Version: e111_eg58_p18_f463989d, January 2024<br>Parameters: (i) organism: Homo sapiens (Human), (ii) target namespace: HGNC, (iii) numeric IDs treated as: GeneCards_acc))
**For gene annotations that were not identified in g:Convert, further annotation searches were performed using:**
GeneCards (Version: 5.20, April 2024)
Uniport (Version: 2024_03, May 2024)
Ensembl (Version: 112, May 2024)
**microRNAs**
microRNAs were searched in GeneCards and were converted into their miRbase IDs including the strand from which they originate
**circRNAs**
circRNAs were searched in circBase to identify genes associated with circBase identifiers (i.e hsa_circ_0017543) (33,34) and the associated genes were then entered in g:Convert. For circular RNA (circRNA), the final annotation used in the analysis was the converted gene name using a format that includes the word "circ" to indicate its circular origin. For example, if the circRNA is derived from the gene ETFA, it would be annotated as "circETFA".

ESM Table 5: Studies investigating placental protein abundance in diabetes

	Ashraf et al., 2019	Assi et al., 2020	Burlina et al., 2019	Chen et al., 2023	Ge et al., 2023	Hu et al., 2022	Lapolla et al., 2013	Liu et al., 2012	Majali-Martinez et al., 2021	Roverso et al., 2016	Wei et al., 2022	Zaugg et al., 2020
**Type of diabetes**												
GDM												
T1DM												
**Sample**												
Placenta												
Uncomplicated (n)	7	5	12	5	6	6	20	8	11	6	5	11
Diabetes (n)	7	5	13	5	6	6	20	8	12	5&6*	5	11
Treatment	D	N/A	D	N/A	N/A	N/A	D	D	N/A	D	N/A	3D, 8I
First trimester												
Third trimester												
**Methods**												
Gel-based proteomics												
Label-free proteomics												
Label proteomics												
Protein array												
**Proteins identified**												
Total protein in gel-free methods	N/A	2103	160	3695	3633	5332	N/A	N/A	60	159	4609	N/A
Spots identified in gel-based methods	N/A						N/A	1000		1000		
Selected spots	4						N/A	21		26		
**Differentially abundant proteins (n)**												
	2	64	3	123	68	114	10	15[a]	4	81[b]	37	2
**Full list of identified protein changes available for analysis**												
Yes												
**Number of unique protein changes in diabetic placentas**												
	2	64	3	123	68	114	10	13[a]	4	41[b]	37	2
**Regulation in diabetes**												
# higher level in diabetes	2	40	2	100	21	36	0	9	0	21,5[c]	12	2
# lower level in diabetes	0	24	1	23	47	78	10	4[a]	4	19,5[c]	25	0

GDM: gestational diabetes mellitus, T1DM: type 1 diabetes mellitus, N/A: not available/applicable, D: diet, I: insulin, *six samples in gel-based proteomics and five samples in label-free proteomics, [a]three different spots identified the same protein with lower abundance in diabetes (six total proteins with lower abundance, but four unique), [b]multiple spots identified for some proteins, [c]ALB was found in both higher and lower abundance lists.

ESM Table 6: Studies investigating placental RNA abundance in diabetes

	Bao et al., 2022	Bari et al., 2016	Chen et al., 2022	Ding et al., 2018	Enquobahrie et al., 2009	Guiyu et al., 2022	He et al., 2023	Inno et al., 2021	Jiao et al., 2023	Kang et al., 2022	Kedziora et al., 2022	Lekva et al., 2016	Li et al., 2015	Li et al., 2018	Li et al., 2022	Lu et al., 2022	Magee et al., 2014	Ort et al., 2005	Radaelli et al., 2003	Radaelli et al., 2009	Sober et al., 2015	Steyn et al., 2019	Tang et al., 2020	Wang et al., 2019	Wang et al., 2021	Xu et al., 2017	Yan et al., 2018	Yang et al., 2021	Zhang et al., 2021	Zhao et al., 2011
**Type of diabetes**																														
GDM	■	■	■	■	■	■	■	■	■	■	■	■	■	■	■	■	■	■	■	■	■	■	■	■	■	■	■	■	■	■
T1DM											■									■										
T2DM											■																			
**Sample *(n**; GDM, n; T1DM, n; T2DM)																														
Placenta	■		■	■	■	■	■	■		■	■	■	■	■	■	■	■	■	■	■	■	■	■	■	■	■	■		■	■
Isolated cells		■							■																			■		
Controls (n)	3	4	4	8	21	4	3	8	5	3	29	10	5	3	3	20	5	8	8	5	8	6	3	3	4	3	3	2	3	2
Diabetes*	3	4	4	8	19	4	3	8	5	6	12, 17, 3	10	5	3	4	21	5	12	7	9, 6	8	6	3	3	4	3	3	2	3	2
Treatment*	N/A	I	D	N/A	9D &10I	N/A	N/A	N/A	C	NI	OMd	NI	N/A	OM	N/A	N/A	4D & 1M	5D & 7D-I	I	I, N/A	N/A	N/A	N/A	N/A	N/A	N/A	N/A	NI	D&I	N/A
Second trimester													N/A	N/A												N/A				■
Third trimester	■	■	■	■	■	■	■	■	■	■	■	■	N/A	N/A	■	■	■	■	■	■	■	■	■	■	■	N/A	■	■	■	■
**Methods**																														
Microarray		■			■	■							■	■			■		■	■						■				■
RNA-sequencing	■		■	■			■	■		■	■	■			■	■					■	■	■	■	■		■		■	
scRNA-seq									■																			■		
GeneCalling																		■												
**RNA**																														
circRNA	■																						■	■			■			

miRNA				■				■		■	■		■	■									■			■			■	
mRNA or/and ncRNA		■	■	■	■	■	■		■	■	■	■			■	■	■	■	■	■	■	■	■		■			■		■
**Transcripts/probes identified**																														
	4,955	28,869	16,473	27,657[b]	14,453	N/A	33,322	417	N/A	N/A	N/A	N/A	N/A	N/A	N/A	N/A	94	1,441	2,682	N/A	22,896	N/A	17,130[i]	8,321	12,935	N/A	48,270	N/A	764	N/A
**Differentially abundant RNA (n;GDM , n;T1DM, n;T2DM)**																														
	37	46	269	250	66	4,883	969	0	N/A	757	0, 0, 93	0	29	N/A	725[f]	1,442	20	N/A	435	35, 26	4	1,489	432[i]	46	360	43	482	N/A	157	243
**Full list of identified protein changes available for analysis**																														
Yes	■	■		■	■			■		■	■	■	■			■	■			■	■		■	■	■	■		■		■
No (n)			■			■	■		■					■	■			■	■			■					■		■	
**Number of unique RNA changes in diabetic placentas (available data)**																														
	36[a]	46	24	313[b]	66	1	4	0	23	757	93	0	28[e]	1	40[f]	1,439[g]	20	70[h]	120	35, 26	4	51	422[i]	44[j]	360	43	37[k]	34[l]	14	240[m]
**Regulation in diabetes (available data)**																														
# higher level in diabetes	19	17	81	213[b]	66	1	0	0	12	324	75	0	3	1	20[f]	1001	13	38	254	30, 24	4	23	205[i]	3	64	28	19	19	8	69
# lower level in diabetes	18	29	188	100[b]	0	0	4	0	11	433	18	0	25	0	20[f]	441	7	32	181	5, 2	0	28	217[i]	41	296	15	18	15	6	174

[a]two circRNA from PRKAR1B were identified, both downregulated in GDM, [b]transcripts/probes identified: mRNAs & lncRNAs:26,255, miRNAs:1,402; differentially abundant RNA: mRNAs: 281, miRNAs:32 ; number of unique RNA changes in diabetic placentas (available data); mRNAs & lncRNA: 281, miRNAs:32; regulation in diabetes (available data): mRNAs & lncRNAs: 204, miRNAs:9 (higher level) and mRNAs & lncRNAs: 77, miRNAs: 23 (lower level), [d] GDM: 12 (5 OM), T1D: 17 (15 OM), and T2D:3 (3 OM), [e]miR-30d two times on the list, [f]differentially abundant RNA: mRNA:647 & lncRNA:78; number of unique RNA changes in diabetic placentas (available data): mRNA:20 & lncRNA:20; regulation in diabetes (available data): mRNA:10 & lncRNA:10 (higher level) and mRNA:10 & lncRNA:10 (lower level), [g] some duplicates, [h] genes were differencially expressed in GDM samples treated with diet compared to uncomplicated samples, but where not significant in the insulin-treated GDM samples compared to uncomplicated samples, [i]transcripts/probes identified: mRNAs & lncRNAs: > 48,000, circRNA: 9,513 & miRNAs: 2,817; differentially abundant RNA: mRNAs & lncRNAs: 315 circRNA: 114 & miRNAs: 4; number of unique RNA changes in diabetic placentas (available data); mRNAs & lncRNAs: 314 (HLA-B as duplicate), circRNA: 105 (6 N/A and 2 circRNA from the same gene upregualted and 2 circRNA from same gene downregulated , 1 circRNA in both upregulated and downregulated list= 9 duplicates and N/A) & miRNAs: 4; regulation in diabetes (available data): mRNAs & lncRNAs: 152+0.5 (HLA-B in both up/downregulated lists), circRNA: 50+0.5 (circCAMSAP1 both up/downregulated) & miRNAs:2 (higher level) and mRNAs & lncRNAs: 162+0.5 (HLA=B), circRNA: 54+0.5 (circCAMSAP1) & miRNA:2; [j]circRNAs from the same gene with same regulation (circTMEM184B and circPAPPA2); [k]circRNAs from the same gene with same regulation (circGCN1L1 and circFNBP4)); [l]differentially expressed genes in multiple trophoblast clusters with same directionality(upregualted: FN1, HSPA1A, HSPA1B, HSPA6, IFIT2 and downregualted: DEFA3, S100A8, S100A9), [m]duplicates with same directionality (*GNLY*, *HSD11B1*,*COL17A1*). N/A: not available, I: insulin, D: diet, C: combination of diet and medication, NI: no treatment with insulin, OM: on medication (not specified), M: metformin.

ESM Table 7: GO&FEA of genes with agreement in regulation direction of RNA and protein abundance.

Biological process	REACTOME
positive regulation of biological process (0.45, <0.05)	response to elevated platelet cytosolic Ca2+ (6.16, <0.001)
regulation of multicellular organismal process (0.61, <0.05)	platelet activation, signaling and aggregation (3.11, <0.001)
regulation of body fluid levels (1.89, <0.05)	platelet degranulation (6.4, <0.001)
positive regulation of response to stimulus (0.75, <0.01)	
response to stress (0.6, <0.01)	
positive regulation of cellular process (0.46, <0.05)	
cell adhesion (1, <0.001)	
wound healing (2.12, <0.001)	
response to wounding (1.8, <0.001)	
hemostasis (2.98, <0.01)	
coagulation (3.44, <0.001)	
blood coagulation (3.08, <0.01)	
platelet activation (3.71, <0.05)	
platelet aggregation (5.48, <0.05)	

Terms associated with 47 genes with same directionality (pathway enrichment factor (%), adjusted p-value)

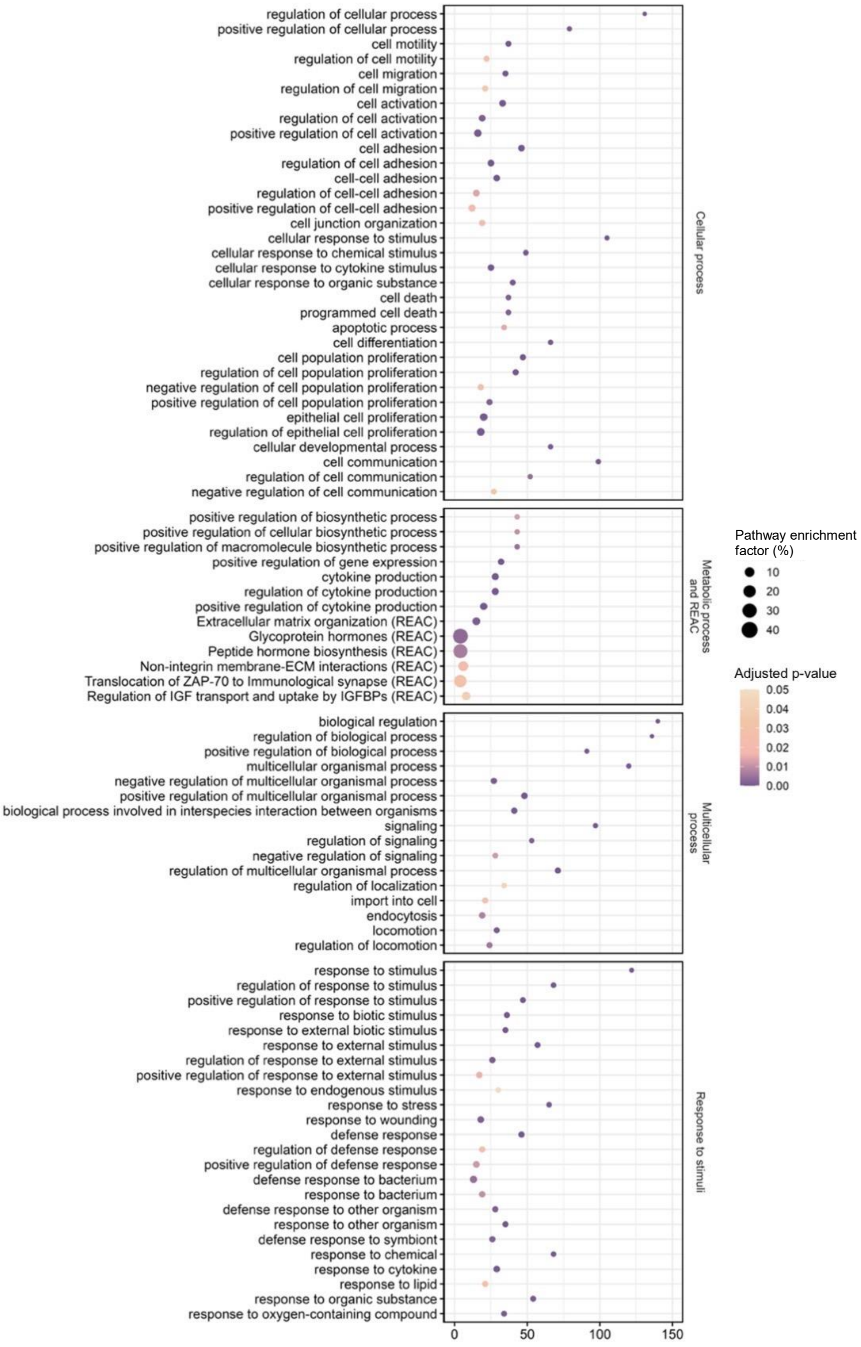

ESM Figure 1: Further FEA terms of common differentially abundant RNAs

Further FEA terms associated with the 189 RNAs with consistent directionality changes across studies. The size of bubbles represents the pathway enrichment factor (percentage of intersection size divided by term size), with larger bubbles indicating a higher enrichment factor. The x-axis reflects the number of genes (representing RNAs) in the query annotated to each term. Bubble colour indicates adjusted p-value, with darker colour representing lower adjusted p-value.

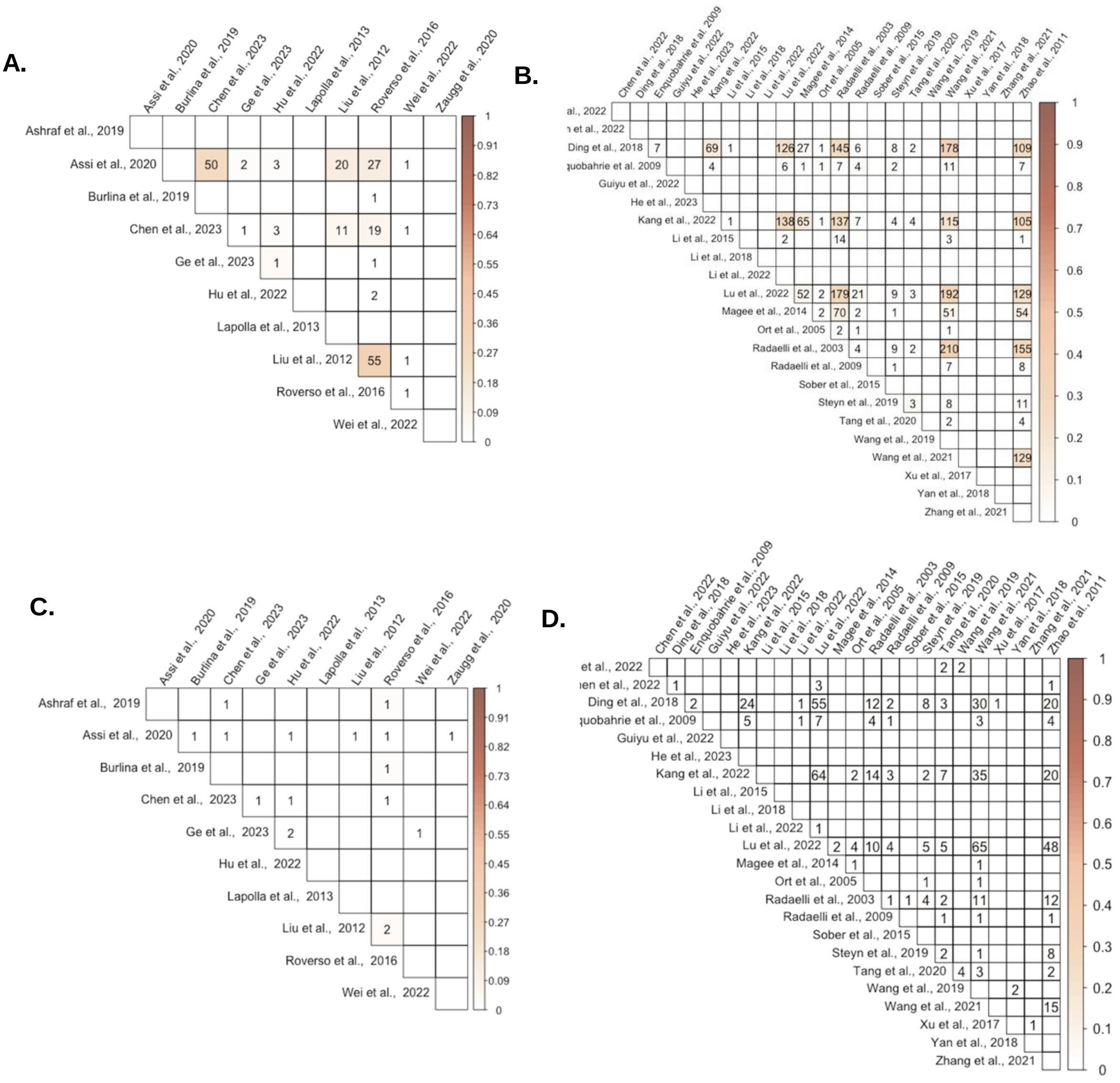

ESM Figure 2: Higher similarity score between shared FEA terms compared to individual protein and RNAs

Pairwise comparisons revealed a high number of shared terms using the individual list from (**A**) protein and (**B**) RNA studies with higher similarity coefficient compared to shared (**C**) proteins or (**D**) RNAs between studies. The number inside each box indicates the pairwise intersections between studies and the colour of each box, reflects the Jaccard index, which ranges from zero (low similarity; no shared genes or terms) to one (high similarity; all genes or terms found in both studies / identical studies).

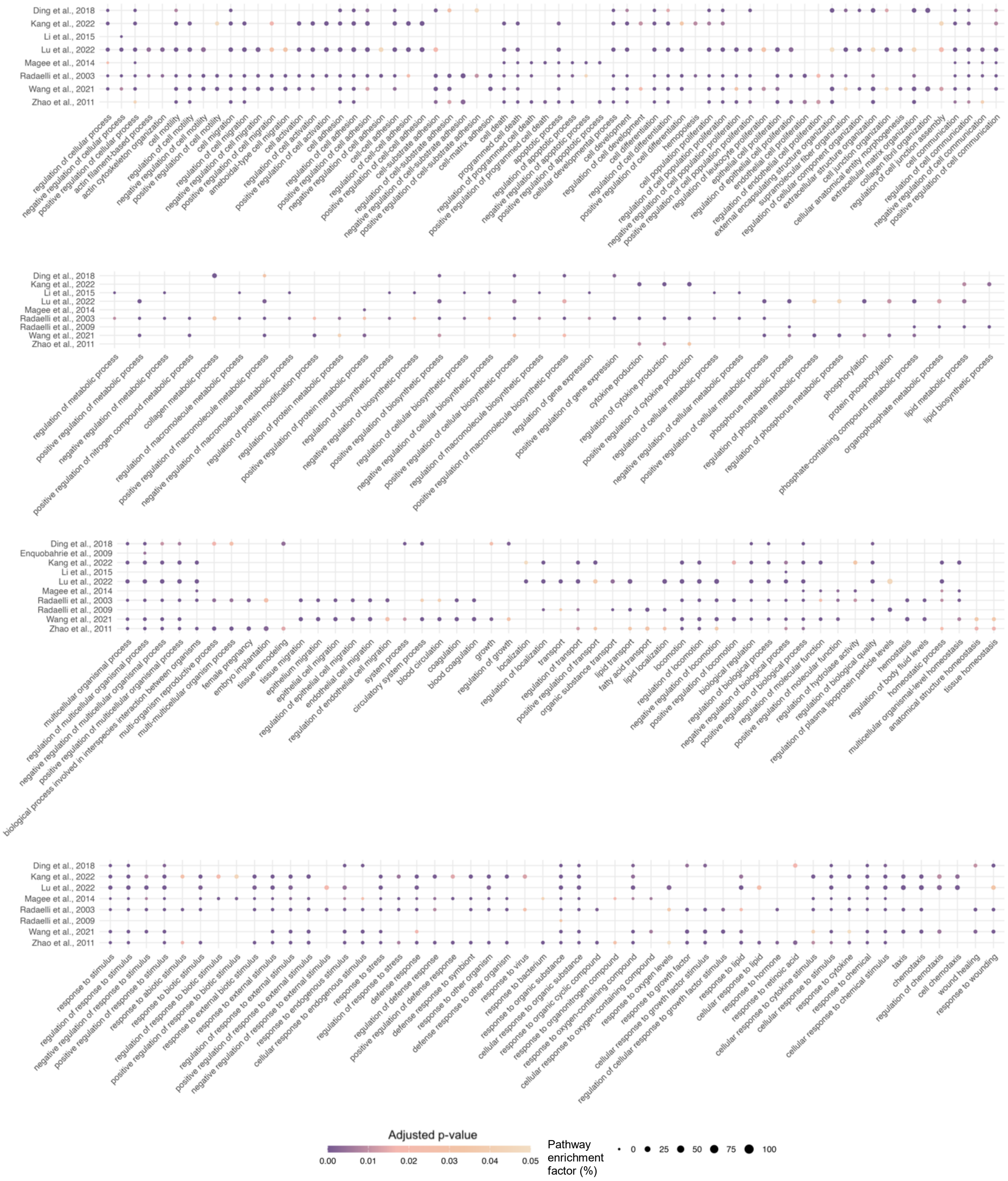

ESM Figure 3: Further FEA terms of all individual RNA studies

The y-axis shows the study in which each FEA term (x-axis) was identified. Bubble size represents the pathway enrichment factor (percentage of intersection size divided by term size), with larger bubbles indicating a higher enrichment factor and colour indicates adjusted p-value, with darker colour representing lower p-value.

-

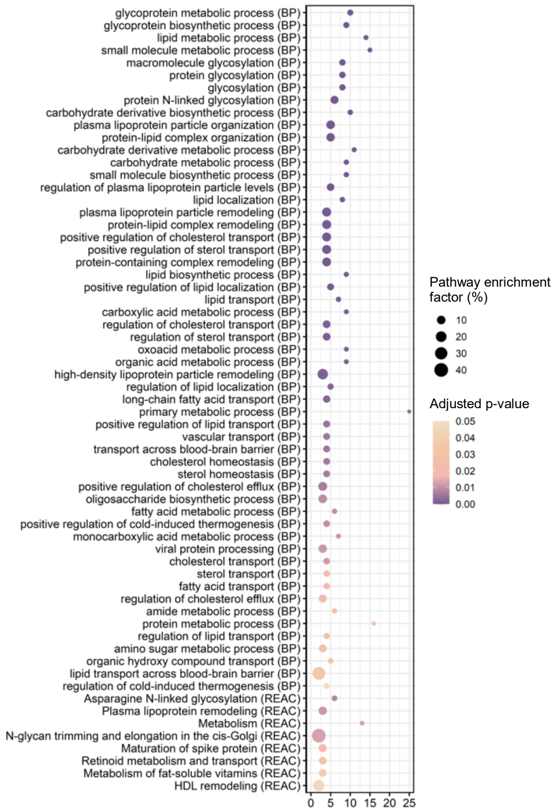

B.

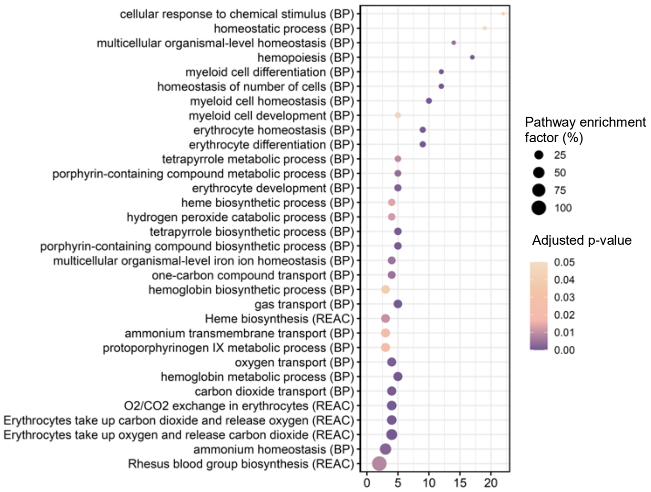

C.

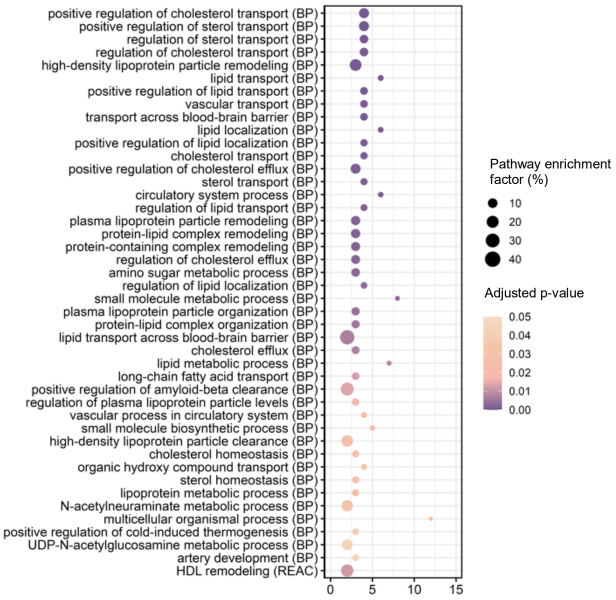

ESM Figure 4: FEA terms of differentially abundant RNAs in type 1 and type 2 diabetes and of shared RNAs with consistent directionality changes across GDM studies

(**A**) FEA terms of 26 RNAs that are differentially abundant in type 1 diabetes compared to uncomplicated placentas. (**B**) FEA terms of 34 RNAs that are

differentially abundant in type 2 diabetes compared to uncomplicated placentas. (**C**) FEA term of 14 differentially abundant RNAs with consistent directionality changes across type 1 diabetes mellitus compared to uncomplicated placentas and GDM compared to uncomplicated placentas. The size of bubbles represents the pathway enrichment factor (percentage of intersection size divided by term size), with larger bubbles indicating a higher enrichment factor. The x-axis reflects the number of genes (representing RNAs) in the query annotated to each term. Bubble colour indicates adjusted p-value, with darker colour representing lower adjusted p-value. BP; biological process, REAC; REACTOME pathways.
